# Diversity of bacterial community during ensiling and subsequent exposure to air in whole-plant maize silage

**DOI:** 10.5713/ajas.17.0860

**Published:** 2018-04-25

**Authors:** Zongfu Hu, Jie Chang, Jianhua Yu, Shuguo Li, Huaxin Niu

**Affiliations:** 1 College of Animal Science and Technology, Inner Mongolia University for Nationalities, Tongliao 028000, China; 2 Inner Mongolia Key Laboratory of Toxicant Monitoring and Toxicology, Tongliao, China

**Keywords:** High-through Sequence, Bacterial Community, Diversity, Lactic Acid Bacteria, Whole-plant Maize Silage

## Abstract

**Objective:**

To describe in-depth sequencing, the bacterial community diversity and its succession during ensiling of whole-plant maize and subsequent exposure to air.

**Methods:**

The microbial community dynamics of fermented whole-plant maize for 60 days (sampled on day 5, 10, 20, 40, 60) and subsequent aerobic exposure (sampled on day 63 after exposure to air for 3 days) were explored using Illumina Miseq sequence platform.

**Results:**

A total of 227,220 effective reads were obtained. At the genus level, there were 12 genera with relative abundance >1%, *Lactobacillus*, *Klebsiella*, *Sporolactobacillus*, *Norank-c-cyanobacteria*, *Pantoea*, *Pediococcus*, *Rahnella*, *Sphingomonas*, *Serratia*, *Chryseobacterium*, *Sphingobacterium*, and *Lactococcus*. *Lactobacillus* consistently dominated the bacterial communities with relative abundance from 49.56% to 64.17% during the ensiling process. *Klebsiella* was also an important succession bacterium with a decrease tendency from 15.20% to 6.41% during the ensiling process. The genus *Sporolactobacillus* appeared in late-ensiling stages with 7.70% abundance on day 40 and 5.32% on day 60. After aerobic exposure, the *Lactobacillus* decreased its abundance from 63.2% on day 60 to 45.03% on d 63, and *Klebsiella* from 5.51% to 5.64%, while *Sporolactobacillus* greatly increased its abundance to 28.15%. These bacterial genera belong to 5 phyla: Firmicutes (relative abundance: 56.38% to 78.43%) was dominant, others were Proteobacteria, Bacteroidetes, Cyanobacteria, and Actinobacteria. The bacterial communities clearly clustered into early-ensiling (d 5), medium-ensiling (d 10, d 20), late-ensiling (d 40, d 60), and aerobic exposure (d 63) clusters, with early- and late-ensiling communities more like each other than to the aerobic exposure communities.

**Conclusion:**

High-throughput sequencing based on 16S rRNA genes proved to be a useful method to explore bacterial communities of silage. The results indicated that the bacterial communities varied during fermentation and more dramatically during aerobic exposure. The study is valuable for understanding the mechanism of population change and the relationship between bacteria and ensilage characteristics.

## INTRODUCTION

The rapid growth of meat and milk production in China requires development of an efficient silage industry. Maize straw is an important feed resource for ruminants. In the north of China, such as Inner Mongolia, it is not only a main base for milk, beef and mutton production, but also a main production base of maize. Many of silage maize variety are cultivated for ruminants feed.

Ensiled silage has the benefit of preventing loss in nutrition value and can keep well throughout a year or more. The essence of the silage is that lactic acid bacteria (LAB) multiply in the anaerobic environment and produce lactate which drops the pH to below 4.0. The low pH in turn prevents or restrains the growth of other infectious microbes.

Microbial succession in food fermentation or silage has been previously studied. For instance, Zheng et al [1] examined microbial community dynamics of ensiling alfalfa *Medicago sativa* and found that *Lactobacillus* dominated the whole process of ensiling like the dynamics patterns of ensiling reported by McGarvey et al [2]. Li et al [3] reported the bacterial community succession in *Vicia faba* fermentation persisted for 77 days using MiSeq analysis base 16S rRNA genes. Yang et al [4] used 16S rRNA sequences to report the bacterial community diversity and dynamics for 15 days fermentation of douchi sauce. For maize ensiling, Lin et al [5] reported dominant establishment of *lactobacillus* in early stages of maize ensiling (1 to 7 days). Subsequent studies by Zhou et al [6] also demonstrated the dominant position of *lactobacillus* after 60 days of ensiling in whole-plant corn silage.

However, research on maize silage production has mainly used culture-dependent methods or denaturing gradient gel electrophoresis (DGGE) [5–7], and lack using new techniques such as high-through sequence which can give a full-scale insight into the microbial community without culture-dependent process. In this study, we used Illumina MiSeq to describe the bacterial community dynamics during ensiling of whole-plant maize; this will enlighten the understanding of the dynamic process of bacterial community during the fermentation of maize.

## MATERIALS AND METHODS

### Dealing of samples

Raw maize samples were collected from a local meadow named Chajintai in Kerzuozhong County (E122° 15′, N43° 38′), Inner Mongolia, China, on Oct 8, 2016. The fresh maize was harvested by combine-harvester 20 cm from ground and chopped into 1 to 2 cm in lengths and immediately transported to the laboratory for processing. Three kilograms of the chopped maize material was compressed and sealed in 18 polyethylene silo bags (Hiryu KN type, 400×600 mm, Asahikasei, Tokyo, Japan) using a vacuum sealer (BH 950, Matsushita, Tokyo, Japan) for air removal. The silo bags were stored at room temperature (25°C). Samples were taken from three bags per timing at 5, 10, 20, 40, 60, and 63 days (the bags were opened for exposure to air at day 60 and lasted for 3 days), respectively, and divided into two portions. One part was sampled in 50 mL cryogenic vials and stored in −80°C for 16S rRNA gene amplicons sequencing, and another was stored in −20°C for biochemical analysis.

### Chemical analyses

The dry matter (DM) content of silage samples was determined by drying 100 g of samples collected on each sampling day at 65°C for 48 h in a dry oven [8]. The pH value was measured as follows: 10 g of silage was mixed with 90 mL of deionized water and shaken for 5 min every other 50 min, after 3 h, the pH value of filtrate form silage was measured by electrode pH meter (S20K, Mettler Toledo, Greifensee, Switzerland). Concentrations of lactic acid and acetic acid were measured in high performance liquid chromatography (1100, Agilent, Palo Alto, CA, USA) fitted with a UV detector (210 nm) and a column (ICSep COREGEL-87H, Transgenomic, Inc., New Haven, CT, USA). The mobile phase was 0.005 M H_2_SO_4_ at a flow rate of 0.6 mL/min at 55°C [9]. Fifteen grams of fresh forage or silage at each sampling day was combined with 135 g of de-ionized H_2_O, blended for 30 s and strained through four layers of cheesecloth. The liquid fraction was sampled and analyzed for and ammonia N (NH_3_-N) as described by Zahiroddini et al [10].

### DNA extraction of the microbial community

The fermented maize straw was sampled (20 g for each bag) and quickly frozen at −80°C for DNA extraction. Silage (20 g) was homogenized with distilled water (80 mL), then filtered through two layers of medical gauze and centrifuged for 15 min at 10,000 g at 4°C.

Triplicate samples of centrifuge production were dissolved and blended together. DNA was extracted using the FastDNA SPIN Kit for Soil (MP Biomedicals, Santa Ana, CA, USA), according to the manufacturer’s instructions [11]. The DNA quality was checked by 1% agarose gel electrophoresis and spectrophotometer (NanoDrop2000, Thermo Scientific, Waltham, MA, USA, optical density at 260 nm/280 nm ratio).

### Polymerase chain reaction amplification of target region

Primers 338F (ACTCCTACGGGAGGCAGCA) and 806R (GGACTACHVGGGTWTCTAAT) [12] both with 12 bps barcodes were used to amplify the V3–4 hyper-variable region of the bacterial 16S rRNA gene. The sequences were assigned to their respective samples according to their barcodes of primers. The polymerase chain reaction (PCR) amplification of bacterial 16S rRNA V3–4 region was performed as described in TransStart FastPfu system (Transgen Biotech Company, Beijing, China) [13]. The PCR cycle conditions were the following: an initial denaturation at 95°C for 3 min, 27 cycles of 95°C for 30 s, 55°C for 30 s, and 72°C for 45 s, and a final extension at 72°C for 10 min. Each 20 μL reaction mixture consisted of 4.0 μL of 5×FastPfu Buffer, 2 μL of deoxynucleoside triphosphate mix (2.5 mM each), 0.4 μL of FastPfu DNA Polymerase, 10 ng of template DNA, 0.8 μL of Forward Primer 338F (5 μM), 0.8 μL of Forward Primer 806R (5 μM), 0.2 μL of bovine serum albumin, and others of double-distilled H_2_O.

### Library preparation and sequencing

NEB Next Ultra DNA Library Prep Kit for Illumina (New England Biolabs Inc., Ipswich, MA, USA) was used to generate sequencing libraries according to the manufacturer’s instructions. The library quality was assessed by spectrophotometry and then sequenced on Illumina Miseq PE300 platform (Illumina Corporation, San Diego, CA, USA) at the Shanghai Majorbio Bio-Pharm Technology co., Ltd. (Shanghai, China).

### Bioinformatics analysis of sequencing data

The operational taxonomic units (OTUs) clustering used Usearch (vsesion 7.1 http://drive5.com/uparse/) [14] at a 97% identity threshold to create OUTs table. The OUTs representing sequences were compared with data online Silva (Release123 http://www.arb-silva.de) and used RDP classifier Naive Bayes (version 2.2 http://sourceforge.net/projects/rdp-classifier/) to obtain species taxonomy information [15].

Rarefaction curves and alpha-diversity indices were calculated by software Mothur (version v.1.30.1 http://www.mothur.org/wiki/Schloss_SOP#Alpha_diversity) [16]. The cluster analysis of sample hierarchy used Qiime platform (http://qiime.org/scripts/assign_taxonomy.html), R language was used to determine beta diversity. Venn figures were calculated by R language, and Heat maps analysis was performed by R language vegan package [17].

### Statistical analysis

All results are reported as the mean±standard error of three replicate groups, and data were subjected to one-way analysis of variance. When there were significant differences (p<0.05), the group means were further compared with Duncan’s multiple range tests. All statistical analyses were performed using SPSS 17.0 (SPSS, Chicago, IL, USA).

## RESULTS

### Chemical analysis of whole-plant maize ensiling

The DM content of silages slightly declined during ensiling. The pH dropped from 4.10 in sample d 5 to about 3.8 in d 10 to d 60 samples, and slightly increased to 3.91 after exposure to air for 3 days (Table 1). The lactic acid content showed increasing tendency during ensiling. Silage from d 20 to d 63 samples had higher lactic acid content than d 5 to d 10 samples. Compared to d 60, the lactic acid content of sample d 63 showed a non-significant decrease. Acetic acid at low level in d 5 and d 10 samples, significantly increased to about 16.80 mg/g DM in d 40 and d 60 samples, and then increased remarkably to about 23.78 mg/g DM in d 63 sample. The ammonia nitrogen slightly increased during ensiling and subsequently during aerobic exposure. The level of ammonia nitrogen in d 5, d 10, and d 20 samples was lower than sample d 40, d 60, and d 63.

### Sequence analysis and alpha-diversity of bacterial community

A total of 227,220 valid reads were obtained from the 6 merged samples (Table 2), with an average of 37,870±6,425 reads per sample, and the mean length of the target sequences were 447.5 bps (Table 2). After quality control, the sequence with chimera and single sequence OTUs were eliminated, and the high quality sequences of 206,842 were obtained, with an average of 34,490±7,515 reads per sample. For limiting the sampling error, we sub-sampled all of the six samples to 29,300 sequences. All these reads were clustered into 357 OTUs based on 97% sequence identity, and these OUTs were assigned to 295 species, 207 genera, 115 families, 71 orders, 34 classes, and 17 phyla. The OTUs number with abundance >1% were at the range of 9 to 15 different with samples (Table 2). With the number of samples increasing, the number of OUTs in all samples increased from about 262 to 357, while the core OUTs decreased from 261 to 146 with a ratio of 40.9% (146/357). Richness accumulated observed (Sobs) and abundance based coverage estimator (Ace) showed the slight increase in richness of the bacterial community in ensiling [4,18]. Good’s coverage of all the samples was above 0.99 [1], the curves of Shannon and rarefaction flattened [2], indicating that the sequence quantity was adequate and covered almost all of bacteria in the samples.

### Cluster analysis of bacterial community

**Figure 1:**
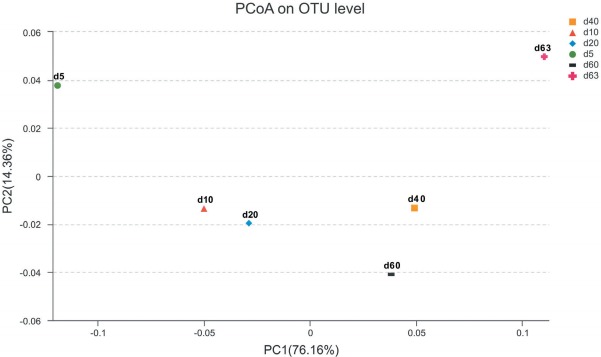
PcoA analysis of the silage bacterial community based on Weighted Unifrac distance. The Weighted Unifrac distance considers both the presence/absence of OTUs and relative abundance of OTUs. Each point represents a silage sample with different color and shape. The first principal component (PC1) is plotted on the X-axis, the second principal component (PC2) is plotted on the Y-axis. PcoA, Principal coordinate analysis; OTUs, operational taxonomic units.

The principal coordinate analysis based on weighted unifrac provided four clusters clearly separated in relation to the ensiling time (Figure 1). Sample from d 5 were clustered as an early-ensiling cluster. Sample from d 10 and d 20, were clustered as a midterm-ensiling cluster. The third cluster, including samples from d 40 and d 60, were grouped as a late-ensiling cluster. The fourth cluster, including d 63 sample alone was clustered as aerobic exposure cluster.

### Bacterial succession during ensiling process and exposure stage

**Figure 2:**
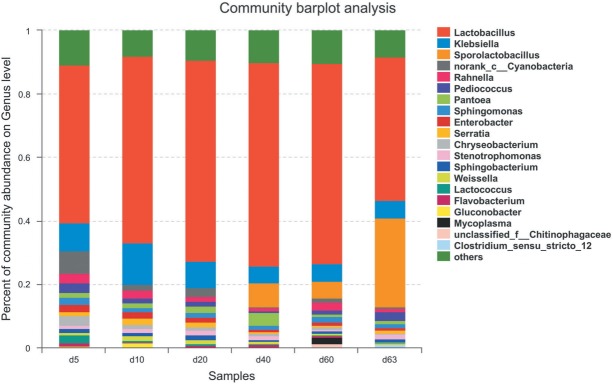
Relative abundance of bacterial community in maize silage during ensiling and air exposure periods at genus level. X-axis represents sample day, Y-axis represents the relative abundance. Genera with abundance lower than 1% in samples were not shown. The genera could not be assigned were marked as ‘unclassified’ or ‘norank’, then they were assigned at family level or up one or more levels.

Changes in the bacterial community during ensiling are presented in Figure 2. At the phylum level, bacterial communities in all samples were dominated by Firmicutes (relative abundance: 57.31% to 78.40%) and Proteobacteria (relative abundance: 31.02% to 16.94%), others were Bacteroidetes (relative abundance: 6.48% to 2.26%), Cyanobacteria (relative abundance: 6.97% to 1.23%), Actinobacteria (relative abundance: 1.14% to 1.98%), Chloroflexi.

At genus level, there were 12 genera shared by all of merged samples with >1% relative abundance, namely *Lactobacillus*, *Klebsiella*, *Sporolactobacillus*, *C-cyanobacteria*, *Pantoea*, *Pediococcus*, *Rahnella*, *Sphingomonas*, *Serratia*, *Chryseobacterium*, *Sphingobacterium*, and *Lactococcus* (Figure 2).

At the early stage of ensiling in sample d 5, the most abundant reads were *Lactobacillus* (49.57%), followed by *Klebsiella* (9.03%) (Figure 2). The class Cyanobacteria has the abundance of 6.97% which could not be classified to genus level. Other genera with >1% abundance were *Chryseobacterium* (3.12%), *Pediococcus* (3.16%), *Rahnella* (3.01%), *Lactococcus* (2.63%), *Sphingomonas* (2.34%), *Enterobacter* (2.26%), *Pantoea* (1.47%), *Serratia* (1.11%), and *Sphingobacterium* (1.3%).

Changes occurred during the ensiling process. In the sample stage of d 10, *Lactobacillus* increased its abundance to 58.62%, and *Klebsiella* increased to 13.34% (Figure 2). Others with >1% abundance also varied or disappeared, such as *Lactococcus*, while some appeared in >1% abundance, such as *Weissella* (1.47%), *Gluconobacter* (1.52%), and *Stenotrophomonas* (1.30%).

During the ensiling process *Lactobacillus* increased in abundance to at 64.1% in the d 40 sample (Figure 2). *Klebsiella* decreased to 5.29% in the d 40 sample. Conspicuous changes appeared in *Sporolactobacillus*, which first occurred in the d 40 sample at 7.70% abundance and 5.32% in the d 60 sample. At the d 40 stage, the samples had the highest level of LAB *Lactobacillus*, and the lowest genera number (7 genera: *Lactobacillus*, *Klebsiella*, *Stenotrophomonas*, *Sporolactobacillus*, *Flavobacterium*, *Pantoea*, and *Sphingomonas*) with >1% abundance. At d 60 stage, the genus *Lactobacillus* had slightly decreased in abundance (63.2%) as well as *Klebsiella* (5.51%). *Mycoplasma* first occurred with abundance of 2.02%. The class Cyanobacteria which disappeared in d 40 occurred again in d 60.

The 3-day aerobic exposure of the d 60 samples (d 63), greatly impacted the ensiling. The genus *Lactobacillus* decreased to 45.03%, the decrease proportion was up to 29.76% compared to d 60 (Figure 2). The genus *Sporolactobacillus* greatly increased to 28.15%, the increase proportion was up to 429.14% compared to d 60. The genus *Klebsiella* stabilized at an abundance of 5.6%. Other genera with low abundance at this stage were *Sphingomonas* (1.16%), *Pediococcus* (2.88%), *Rahnella* (1.13%), *Serratia* (1.09%), and *Stenotrophomonas* (1.43%).

The most concern in silage is the appearance of harmful bacteria, such as *Clostridium* and *Mycoplasma*. During the ensiling process, there was good control of these harmful bacteria, *Mycoplasma* only appeared in the d 60 sample with an abundance of 2.02% and no *Clostridium* ever appeared. But at the aerobic exposure stage, the *Clostridium* appeared at an abundance of 1.06% (Figure 2).

### The core bacterial community during ensiling process and exposure stage

**Figure 3:**
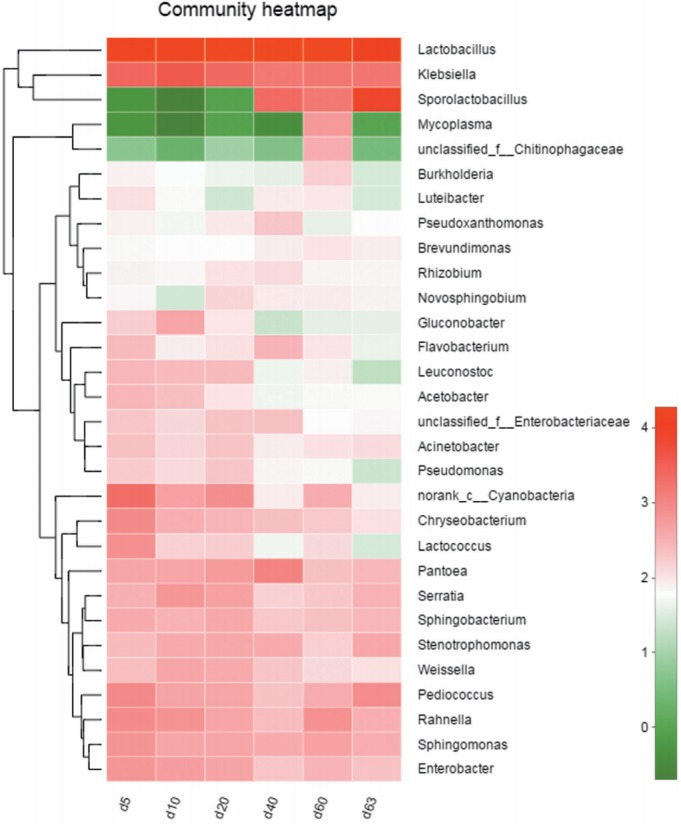
Heatmap analysis of top 30 genera according to relative abundance. X-axis represents sample name, Y-axis represents the genus. Block with red color indicates the high abundance of the genus, the green block indicates the low abundance of the genus. The Hierarchical clustering tree appears on the upside of block while the phylogenetic tree appears on the left side of block.

There were 146 core bacterial species (OTUs) existing in all samples from the ensiling process to the aerobic exposure stage, occupying the proportion of 40.89% of all 357 OTUs. At genus level, they were *Lactobacillus*, *Klebsiella*, *Pediococcus*, *Sphingomonas*, *Serratia*, *Rahaella*, *Pantoea*, *Sphingobacterium*, *Stenotrophomonas*, *Weissella*, *Chryseobacterium*, and *Acinetobacter*, et al. The occurrence of core bacterial community also can be demonstrated by Heatmap analysis (Figure 3).

**Figure 4:**
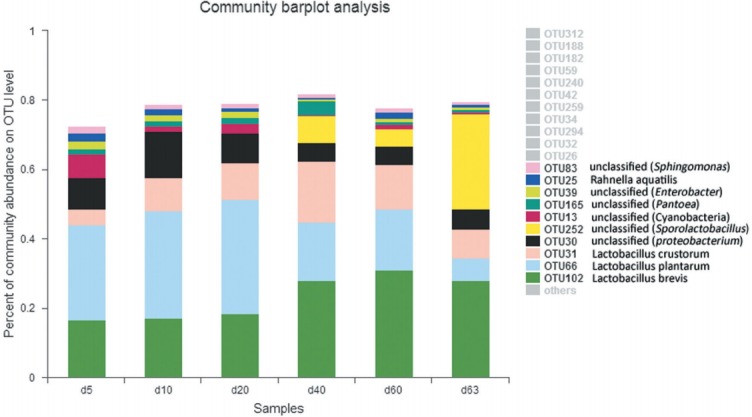
Relative abundance of bacterial community in maize silage during ensiling and air exposure periods at OTUs level. The most 10 common OTUs in all samples were showed. X-axis represents sample day, Y-axis represents the relative abundance. OTUs with abundance lower than 1% in samples were not shown. The species could not be assigned were marked as ‘unclassified’, then they were assigned at genus level or up one or more levels. OTUs, operational taxonomic units.

**Figure 5:**
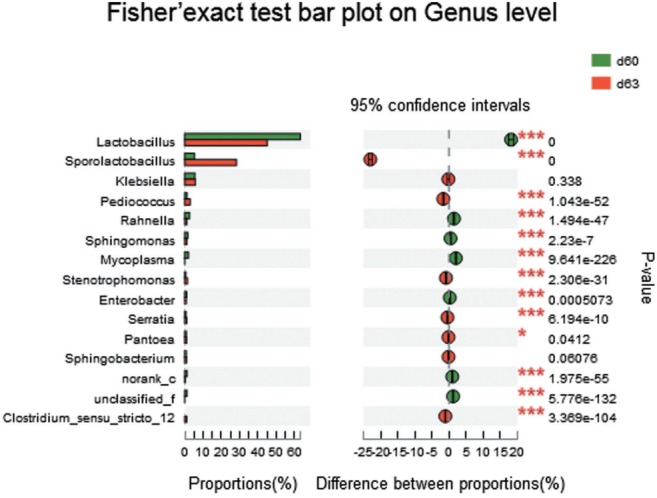
The comparison of silage bacterial species between end of ensiling sample (d 60) and aerobic exposure sample (d 63). Fisher’s two-tailed tests were used to examine the difference of abundance between two samples. The X-axis in column diagram in the left indicates the abundance proportion of each bacterial species, and Y-axis represents bacterial species at genus level. The scatter diagram in the right indicates the difference proportion of species abundance between two samples at 95% confidence interval. * p<0.05, ** p<0.01, *** p<0.001.

The bacterial *Lactobacillus* showed its persistent domination at all stages with abundance 49.57%, 58.62%, 63.24%, 64.11%, 63.20%, and 45.03% in samples d 5, d 10, d 20, d 40, d 60, and d 63 respectively (Figure 2), increasing during ensiling, but greatly decreased at the aerobic exposure stage (d 63) (p<0.001) (Figure 5). The bacteria *Lactobacillus* consisted of *L. plantarum*, *L. brevis* and *L. crustorum* mainly, but the abundance of *L. plantarum* reduced after aerobic exposure (Figure 4). The abundance of *Klebsiella* was 9.03%, 13.34%, 8.04%, 5.29%, 5.51%, and 5.64% in samples d 5, d 10, d 20, d 40, d 60, and d 63 respectively (Figure 2), increasing at the early stages (d 5 to d 10) and decreasing at the later stages. *Sporolactobacillus* occurred at the late stages of ensiling and exposure stage with abundance of 7.7% (d 40), 5.32% (d 60), 28.15% (d 63), the increase at exposure stage was highly significant (p<0.001) (Figure 5).

## DISCUSSION

Compared to extensive work with culture-dependent methods and LAB-focusing [19–21], advanced molecular techniques could better demonstrate the bacterial diversity of maize silage. The present study examined the bacterial diversity of whole-plant maize silage during fermentation (for 60 days) and subsequent aerobic exposure (from d 60 to d 63) by Illumina Miseq sequence platform.

As known from previous studies, 5 to 7 days is a good time to check the microbial composition and biochemical index [5,6]. This was verified by the pH value which reached below 4.0 and the rapid growth of *Lactobacilus* in silage after 5 days of fermentation. For 60 days of fermentation, the pH remained below 4.0 stably and even after opening the package for aerobic exposure. The low pH value insured the anaerobic stability and eventually contributed to the favorable fermentation of whole-plant maize. Lactic acid and acetic acid, produced by LAB, were the reason for pH decline [22]. In the present, the lactic acid increased significantly after 20 days of ensiling, while the significant rise of acetic acid occurred at later stage and aerobic exposure stage. Both acids have benefits for the control of spoilage yeasts or other pathogenic microorganisms [23]. During the ensiling, the content of DM dropped slightly. The content of lactic acid and acetic acid was at a high level, and the ammoniac nitrogen content was in limit ranges. All these indexes showed the excellent fermentation characteristics of the silage.

High-through sequencing has the benefit of finding more bacterial species in silage. We found 295 species belonging to 207 genera in the silages. Except 5 genera of LAB, more bacteria belong to non-LAB. It’s worth noting that previous research mainly focused on LAB, or pathogenic bacteria [5,6], and hardly on others which may play an equally important role during the ensiling process.

It has been well documented that the high content of LAB, such as *Lactobacilus*, is an assurance of rapid decline in pH, which is a key factor ensuring fermentation quality during the ensiling process [23,24]. In the study, the species of *Lactobacillus* dominated the bacterial communities during the whole process of ensiling, even after aerobic exposure. The increasing of *Lactobacillus* abundance (from 49.57% in d 5 to 63.20% in d 60) displayed a positive correlation with the increasing of lactic acid (from 48.37 mg/g in d 5 to 96.9 mg/g in d 60) and acetic acid (from 4.79 mg/g to 16.87 mg/g), indicating that the increase of lactic acid and acetic acid was mainly due to *Lactobacillus*. Some other studies also reported the predominance of *Lactobacillus* in silages [1,5,25]. *Lactobacillus* species, such as *L. plantarum*, *L. brevis*, and *L. buchneri*, are extensively used as inoculants in silage [7,21,24,26]. In our study, the sequences in the genus *Lactobacillus* were mainly assigned to *L. plantarum*, *L. brevis* and *L. crustorum*. *L. plantarum*, a homo-fermentative bacteria, produces only lactic acid, and *L. brevis*, hetero-fermentative LAB, produces both lactic and acetic acid. Their stable presence contributes to the rising of lactic and acetic acid during fermentations. Above all, *L. crustorum* was also a main constituted species of *Lactobacillus* during ensiling and played an important role for good fermentation quality of ensilage. Although *L. crustorum* has been widely reported in food fermentation, little has been reported in silage. As reported by Arasu et al [27], *L. crustorum* was isolated from silage, and showed high growth rates and organic acid (lactic acid, acetic acid) production capability. So, *L. crustorum* may be a potential probiotic for use silage.

In addition to *Lactobacillus*, several other acids-producing bacteria appear in the early stages (d 5 and d 10), and decrease or disappear in subsequent periods, namely *Pediococcus*, *Lactococcus*, *Weissella*, and *Leuconostoc*. This situation was demonstrated in several studies [5,25]. *Pediococcus*, *Lactococcus*, *Weissella*, and *Leuconostoc*, hetero-fermentative bacteria, can produce lactic and acetic acid. The low pH caused by these acids is important for initiating the fermentation of silage. These species have a role of creating a suitable environment for the further growth of *lactobacilli* [21,24]. The LAB profiles of our study are like the DGGE analysis of maize silage from Inner Mongolia which reported the dominant of *Lactobacillus* and the appearace of *Lactococcus*, *Leuconostoc*, and *Weissella* after half a year of ensiling from epiphytic bacteria [28], indicating the richness of epiphytic *Lactobacillus* in this region.

*Gluconobacter*, *Acetobacter*, and *Flavobacterium* were found in minor abundance during the early stages of ensilage. *Gluconobacter* and *Acetobacter* were reported by Gharechahi et al [25] in natural fermented maize silages. *Acetobacter*, an acetic acid producing and nitrogen-fixing bacteria, can be found in many plants [29], and may contributes to the pH decline at the early stage of silages. *Flavobacterium* was found in the silages and developed for inoculants because of its ability to produce cellulase [30]. Cellulase can degrade cellulose fiber to monosaccharides, such as hexose and pentose. From this point, water soluble carbohydrates or sugars were not completely illustrated the substrate available for LAB [22].

The genus *Klebsiella*, an epiphytic microbiota, sub-dominated the bacterial community during the entire process of ensiling. Like our findings, other studies have found that *Klebsiella* is present throughout the fermentation process of corn silage [7]. However, according to the report of Ni et al [31], *Klebsiella* was only found in pre-ensiled corn silage, not ensiling process. Most of *Klebsiella* species, belonging to Enterobacteriaceae family, are found to produce 1, 3-propanediol during fermentation such as *Klebsiella pneumoniae* ATCC 8724 [32], but the propanediol and butyric acid produced by *Klebsiella* was not previously considered to be benefit indicators in silage [22]. In addition to the *Klebsiella*, other bacteria, such as *Pantoea*, *Rahnella*, *Sphingomonas*, *Serratia*, *Chryseobacterium*, *Sphingobacterium*, *Stenotrophomonas*, *Acinetobacter*, and *Enterobacter*, were evenly distributed throughout the fermentation process of silages in lower amounts. In fact, the above bacteria were found in various maize and grass silages [25,30,31,33], but our study covered all these species for the first time. This may be related to our study methods, which cover a greater depth and breadth than other methods, or to different epiphytic bacteria of different silages. Among them, *Pantoea*, *Rahnella*, *Serratia*, *Enterobacter*, and *Enterobacter* belong to the family of Enterobacteriaceae which produces not only organic acids but also ethanol. Such as *Enterobacter* and *Rahnella*, they were found to produce high level of alcohol if they were prevalent on pre-ensiled crops [34].

Aerobic exposure had a noticeable impact on the structure of bacterial communities. In the study, the remarkable changes were the declining of *Lactobacillus* (from 63.20% to 45.03%) and the increasing of *Sporolactobacillus* (from 5.32% to 28.15%). The slightly reduction in the concentration of lactic acid may be due to the decrease of *Lactobacillus*. The increases in the acetic acid contents may be due to the high proportion of *L. crustorum* and *L. brevis*, both of which produce acetic acid. As a facultative anaerobic, *Sporolactobacillus*, a spore-forming, gram-positive bacterium, can produce D-lactic acids by homo-fermentation of hexoses [35]. Although the presence of *Sporolactobacillus* in corn silage and its antifungal activities have been reported by Kharazian et al [36], there is still little coverage of the genus in silage. In our study, *Sporolactobacillus* may be contributed to the maintaining of low pH during the aerobic exposure stage, since the silage in this period was at risk of pH rise due to the decreasing of LAB *Lactobacillus*.

Spoilage and pathogenic microorganisms may be present in silage during fermentation process, such as *Clostridium*, Cyanobacteria, and *Listeria* [37]. *Listeria* was not found in our study, but Cyanobacteria appeared in most of the samples (d 5, d 10, d 20, d 60). *Clostridium* appeared in aerobic exposure period. The species of *Clostridium* may be responsible for the production of butyric acid, which can reduce the content of lactic acid, as well as the production of biogenic amines by protein fermentation [7,22]. It may be related to *Clostridium* that biogenic amines increased, and lactic acid slightly decreased in aerobic exposure samples.

The term succession is defined as the biological changes of an ecosystem in a predictable way of species composition shifts [38]. The predictable dynamics of bacterial diversity were shown in our present reflected by the dominance of *Lactobacillus* over the whole time, indicating the good participation of this epiphytic bacterium. This agreed with other studies [5,25], which reported the predominance of *Lactobacillus* in maize silage.

## CONCLUSION

High-throughput sequencing technology revealed the diversity of the bacterial community in whole-plant maize silage. Although the bacterial community profiles varied during ensiling and aerobic exposure, *Lactobacillus* dominated the ensiling process, while *Lactobacillus* and *Sporolactobacillus* dominated the aerobic exposure samples. It indicated that *Lactobacillus* and *Sporolactobacillus* were the major contributors to the excellent fermentation characteristics. Other bacteria were also participated in the fermentation process. The results of this paper provide valuable information for further development and application of bacteria in silage. The underlying relationship between dynamic patterns of silage microbiota and outcome of fermentation needs further extensive studies.

## Figures and Tables

**Table 1:** The fermentation quality and nutrient composition of the whole maize silage1)

Items	d 5	d 10	d 20	d 40	d 60	d 63
DM (% DM)	32.57±3.13^a^	32.73±1.26^a^	31.83±1.68^a^	31.05±0.46^b^	31.73±2.22^a^	31.17±0.76^ab^
pH	4.10±0.41^a^	3.88±0.23^b^	3.82±0.09^b^	3.76±0.42^b^	3.83±0.08^b^	3.91±0.10^a^
LA (mg/g DM)	48.37±2.14^b^	56.55±6.10^b^	88.19±7.35^a^	96.9±3.83^a^	96.9±2.66^a^	90.84±4.27^a^
AA (mg/g DM)	4.79±0.32^c^	5.08±0.02^c^	9.46±1.72^c^	16.53±2.02^b^	16.87±3.27^b^	23.78±4.14^a^
NH_3_-N (mg/g DM)	0.51±0.02^b^	0.59±0.05^b^	0.72±0.05^ab^	0.92±0.02^a^	0.91±0.11^a^	0.97±0.07^a^

DM, dry matter; LA, lactic acid; AA, acetic acid; NH_3_-N, ammonia nitrogen.

1) The sample name d 5 indicates the silage was sample on 5 days after ensiling; the same as d 10, d 20, d 40, d 60, but d 63 was sampled on 3 days after open the silo from d 60, the same as below diagram.

abc Mean values with different superscripts with in a row differ significantly, p<0.05.

**Table 2:** The sequencing index of samples based on 16S rRNA gene1)

Sample	Valid sequence	High quality sequence	OUTs	OUTs>1%	Ace	Coverage	Shannon	Simpson	Sobs
d 5	34,175	30,149	253	12	293.1	0.9984	2.973	0.1224	253
d 10	34,508	31,243	208	15	228.0	0.9990	2.686	0.1555	208
d 20	39,423	35,410	247	14	282.3	0.9987	2.662	0.1646	247
d 40	43,901	38,658	263	9	288.1	0.9987	2.682	0.1490	263
d 60	30,918	29,476	313	11	334.1	0.9986	2.822	0.1508	313
d 63	44,295	42,006	295	9	327.8	0.9992	2.641	0.1702	295

OTUs, operational taxonomic units.

1) Calculations were performed based on the OTU definition at >97% sequence identity.
